# Leisure-time physical activity and risk of disability incidence: A 12-year prospective cohort study among young elderly of the same age at baseline

**DOI:** 10.1016/j.je.2016.11.004

**Published:** 2017-06-09

**Authors:** Takashi Matsunaga, Mariko Naito, Kenji Wakai, Shigekazu Ukawa, Wenjing Zhao, Satoe Okabayashi, Masahiko Ando, Takashi Kawamura, Akiko Tamakoshi

**Affiliations:** a Department of Preventive Medicine, Nagoya University Graduate School of Medicine, Nagoya, Japan; b Department of Public Health, Hokkaido University Graduate School of Medicine, Sapporo, Japan; c Kyoto University Health Service, Kyoto, Japan; d Center for Advanced Medicine and Clinical Research, Nagoya University Hospital, Nagoya, Japan

**Keywords:** Leisure-time physical activity, Disability, Elderly

## Abstract

**Background:**

To clarify the role of physical activity in preventing disability in Japan, we investigated the association between amount of leisure-time physical activity and incidence of disability among the young elderly.

**Methods:**

In the New Integrated Suburban Seniority Investigation (NISSIN) project conducted from 1996 to 2013, we followed 2888 community-dwelling adults aged 64–65 years with no history of cerebrovascular disease for a median follow-up of 11.6 years. Disabilities were defined as follows based on the classifications of the Japanese long-term care insurance system: 1) support or care levels (support levels 1–2 or care levels 1–5); 2) care levels 2–5; 3) support or care levels with dementia; and 4) care levels 2–5 or death. In addition, we also assessed 5) all-cause mortality.

**Results:**

After controlling for sociodemographic, lifestyle, and medical factors, male participants reporting an activity level of 18.1 metabolic equivalent (MET)-hours/week (the median among those with activities) or more had 52% less risk of being classified as support or care levels with dementia compared with the no activity group (hazard ratio 0.48; 95% confidence interval, 0.25–0.94). No significant association was found among women between amount of leisure-time physical activity and incidence of disability.

**Conclusion:**

We identified an inverse dose–response relationship between the amount of leisure-time physical activity and the risk of disability with dementia in men. Therefore, a higher level of physical activity should be recommended to young elderly men to prevent disability with dementia.

## Highlights

• We examined associations between physical activity and disability incidence.
• We quantified amount of leisure-time physical activity.
• A dose–response relationship was found for the risk of disability with dementia.

## Introduction

In developed and developing countries alike, increases in longevity are accompanied by increases in the number of individuals with disability.^1^ Worldwide, from 1990 to 2013, estimated years lived with disability (YLDs) increased 42.3%, from 537.6 million to 764.8 million.^1^ On the other hand, in Japan, the national long-term care insurance (LTCI) system operated by local governments covers 90% of the health care costs for middle-aged and elderly individuals with disability. When the LTCI system was launched in fiscal year (FY) 2000, the number of beneficiaries was 2.56 million. By 2012, this number had more than doubled, reaching 5.61 million from among the total population of 127 million.^2^ Because disability limits social participation, lowers quality of life, and makes it difficult to live independently in the community,^3^, ^4^, ^5^ health care providers should aim to prevent the incidence of disability in elderly people.

Many studies have reported that regular physical activity (total physical activity, including activities of daily life [e.g., working and housekeeping], leisure-time physical activities, and walking) reduced the risk of disability in the elderly.^6^, ^7^, ^8^, ^9^, ^10^, ^11^, ^12^, ^13^, ^14^, ^15^, ^16^, ^17^, ^18^, ^19^, ^20^, ^21^, ^22^, ^23^, ^24^, ^25^, ^26^, ^27^, ^28^, ^29^, ^30^, ^31^, ^32^, ^33^, ^34^, ^35^, ^36^, ^37^, ^38^, ^39^, ^40^, ^41^, ^42^ In addition, some reviews and guidelines have suggested the appropriate activity level for the elderly population.^40^, ^42^, ^43^ For example, a Japanese guideline recommended performing at least 10 metabolic equivalent (MET)-hours/week in physical activity at any intensity for adults aged 65 years or older.^43^ However, a number of issues remain unclear. First, few studies have measured the intensity and duration of physical activity quantitatively.^7^, ^14^, ^17^, ^18^, ^32^ Therefore, the current guidelines for physical activity require validation.^40^ Second, common physical activities vary among populations.^44^ Therefore, even if the frequency and duration of physical activity are similar, the amount may be different. However, in Japan, many studies have only examined the association between general measures of physical activity (e.g., walking time or frequency and total exercise frequency) and incidence of disability.^9^, ^24^, ^27^, ^34^, ^37^ Third, age is one of the most important risk factors for the incidence of disability.^45^ Previous studies have mainly applied multivariate analysis to adjust for the effect of age,^6^, ^7^, ^8^, ^9^, ^10^, ^12^, ^14^, ^15^, ^17^, ^18^, ^20^, ^21^, ^22^, ^24^, ^25^, ^26^, ^27^, ^29^, ^30^, ^31^, ^32^, ^34^, ^35^, ^36^, ^37^, ^38^, ^39^ but it may be difficult to fully control for confounding by age.^46^

Therefore, in this study, we attempted to quantitatively assess leisure-time physical activity (sports and recreational activities) and follow-up long-term disability onset prospectively among the elderly of about the same age in a Japanese cohort.

## Methods

### Study design and participants

This study was conducted as part of the New Integrated Suburban Seniority Investigation (NISSIN) project, a prospective cohort study targeting adults who were approaching 65 years old at baseline (the young elderly). The methods of that project and the characteristics of the participants have been described elsewhere.^47^ The participants were community-dwelling elderly in a suburban area (Nisshin city). All participants were invited to undergo a medical checkup and asked to complete a questionnaire on sociodemographic, lifestyle, comorbidity, and psychological factors.

From 1996 to 2005, 3073 individuals participated in the baseline survey, which was conducted each year in June. We excluded three and one persons who had relocated or had received an LTCI certification before the checkup, respectively. We also excluded 140 persons who had a history of cerebrovascular disease before the checkup and 41 individuals who had relocated or died before the start of the LTCI system (April 1, 2000). Ultimately, 2888 participants were included in the present analysis.

### Informed consent and approval of the study protocol

Informed consent was obtained from all participants before conducting the medical checkups. For the questionnaire-based study, oral consent was obtained using an opt-out approach until 2001, and written consent was obtained using an opt-in approach thereafter. The study protocol was approved by the ethics committees of the Nagoya University Graduate School of Medicine, the National Center for Geriatrics and Gerontology of Japan, the Aichi Medical University School of Medicine, and the Hokkaido University Graduate School of Medicine.

### Exposure assessment

At baseline, participants reported the type, frequency, and duration per episode of their leisure-time physical activity in the previous year. Activity types listed in the questionnaire and their assigned intensity (METs) are as follows: running or jogging: 7.0; swimming: 5.8; calisthenics: 3.8; baseball or softball: 5.0; tennis: 7.3; golf: 4.8; gateball (Japanese croquet): 3.3; and mountain climbing or hiking: 6.5.^48^ For the response of “other types” of activity (non-pre-coded activity), we applied a weighted-average intensity of 3.9 METs based on the data provided in a free comment field because only partial information regarding non-pre-coded activity types (25%) was available. Although the participants who responded “other types” of activity provided the type of activity, only a data file was kept for most of the participants (75%), and the type of “other” activity was not included in this file. Therefore, we calculated the weighted-average intensity for participants whose questionnaires were kept and applied that value to all the participants. We then calculated the amount of activity (MET-hours/week) by multiplying intensity, duration, and frequency. In accordance with existing guideline for physical activity,^42^ we only counted sessions lasting 10 min or longer.

### Covariates

The following demographic variables were considered: year of participation in the study (continuous variable); currently working (yes or no); marital status (married or other [single, divorced, or widowed]); and educational attainment (junior college and higher or high school and lower). Lifestyle variables were: smoking status (never, former, or current); alcohol consumption (men: none, ≤23 g/day, or >23 g/day; women: none or current drinkers); body mass index (BMI; <18.5, 18.5–24.9, or ≥25.0 kg/m^2^); social activity score^49^ (in tertiles; men: ≤25, 26–28, or 29–54 points; women: ≤27, 28–31, or 32–54 points; items on work, sports, and recreational activities were excluded in this study because they were considered as exposure or other covariates); and total walking time per day, including work and housekeeping (<30 min, 30 min – 1 h, 1–2 h, or ≥2 h).

We included hypertension, dyslipidemia, diabetes mellitus, and neuralgia and/or low back pain as comorbidity variables in the analysis. Hypertension was defined as systolic blood pressure ≥140 mm Hg, diastolic blood pressure ≥90 mm Hg, and/or self-reported medication for hypertension. Dyslipidemia was defined as serum low-density lipoprotein (LDL) cholesterol ≥140 mg/dL (estimated using the Friedewald equation if triglycerides <400 mg/dL) or LDL cholesterol ≥170 mg/dL (estimated as total cholesterol minus high-density lipoprotein [HDL] cholesterol if triglycerides ≥400 mg/dL), HDL cholesterol <40 mg/dL, triglycerides ≥150 mg/dL, and/or self-reported medication for hyperlipidemia. Diabetes mellitus was defined as blood hemoglobin A1c ≥ 6.1% (based on the former method of the Japan Diabetes Society, equivalent to ≥6.5% in the National Glycohemoglobin Standardization Program [NGSP]),^50^ fasting plasma glucose ≥126 mg/dL, and/or self-reported medication for diabetes mellitus. All participants underwent health checkups and provided blood samples the morning after an overnight fast. History of neuralgia and/or low back pain was reported as none, cured, under treatment, or leaving; the last two categories were combined because of the small number of subjects in each category. We used the short version of the Geriatric Depression Scale (GDS) as a screening test for depression and regarded six points or higher as probable depression.^51^

### Follow-up and outcomes

We followed-up the participants prospectively for qualification as an LTCI recipient or death from baseline through the end of December 2013. Participants' LTCI certifications were surveyed by the local government of Nisshin city. We identified all-cause mortality using the resident registry.

In Japan, all individuals aged 65 years or older, or those aged 40–64 years who suffer from age-related diseases are eligible for LTCI benefits. When a person applies to his/her municipality for LTCI benefits, an authorized care manager examines his/her physical and mental status using a standardized questionnaire. Severity of dementia is assessed concurrently based on the “Independence Criteria of Daily Living for the Demented Elderly”.^52^ The responses to the questionnaire are processed using computer software, and the time required for care is estimated. Finally, the certification board, which includes medical doctors and nurses, determines the level of long-term care needed based on the estimated time required for care, as well as on comments from the applicant's family physician.^53^

The LTCI certifications consist of the following seven levels: support levels 1–2 and care levels 1–5. Support levels 1 and 2 are defined as “the patient is independent in basic activities of daily living, but requires some assistance in instrumental activities of daily living”, care level 1 as “the patient requires partial assistance in instrumental activities of daily living”, care level 2 as “the patient requires some assistance in basic activities of daily living”, and care level 5 as “the patient requires total assistance in basic activities of daily living and cannot live without assistance”.^54^ In addition to the level of care required, the severity of dementia is graded as follows: independent; I; IIa; IIb; IIIa; IIIb; IV; and M. Level I is defined as “the patient has some dementia, but can live independently at home and in society,” level IIa as “although the patient has a few symptoms or behaviors that disturb daily living, or difficulty in communication outside the home, they can live independently under another's attention,” and level M as “the patient has severe mental symptoms, behavioral and psychological symptoms of dementia, or severe physical disease, and therefore requires special treatment.”^52^

We defined the incidence of disability in the follow-up based on LTCI certification and mortality as follows: i) support or care levels (i.e., support levels 1–2 or care levels 1–5); ii) care levels 2–5; iii) support or care levels with dementia (level IIa or higher); and iv) care levels 2–5 or death. We considered care levels 2–5 as severe disability because participants with care levels 1–5 had significantly lower ADL ability than those with support levels 1–2,^55^ and for consistency over the follow-up period; the former care level 1 was divided into support level 2 and care level 1 in 2008. In addition, we assessed v) all-cause mortality.

### Statistical analysis

Because many of the participants did not engage in any leisure-time physical activity, we divided the participants into the following three groups according to their amount of activity: “no activity group”; “less than or equal to median group”; and “more than median group”. The median was computed only in those with leisure-time physical activity. We tested for statistical differences in background characteristics between groups using the chi-squared test of independence.

Person-years of follow-up were counted from baseline (medical checkup) to LTCI certification, death of any cause, relocation from the study area (Nisshin city), or December 31, 2013, whichever occurred first. We treated death as censoring, except in the analysis that included mortality as an outcome. The LTCI system was launched in April 1, 2000, so we started follow-up from April 1, 2000 for those persons who had participated in the baseline survey until March 2000.

Next, we examined associations between the amount of leisure-time physical activity and incidence of disability or mortality using Cox proportional hazard models. We performed a trend test by entering a variable scored as 0, 1, or 2 for the three groups with increasing physical activity as a single variable in the model. Because the association between amount of physical activity and incidence of disability differed considerably between sexes, we examined associations by sex. We calculated crude hazard ratios (HRs) in model1; HRs adjusted for year of participation, work, marital status, educational attainment, smoking and drinking habits, and BMI in model 2; and HRs adjusted for covariates in model 2 plus comorbidity (hypertension, diabetes mellitus, dyslipidemia, and neuralgia and/or low back pain), GDS scores, social activity, and total walking time per day in model 3.

In addition, we repeated the fully-adjusted Cox regression (model 3) as sensitivity analyses: i) by excluding events within 3 years from baseline; ii) by excluding those who had participated in the study until 1999; and iii) by excluding LTCI certification through March 31, 2002.

All statistical analyses were performed using Stata software (version 13.1; Stata Corp LP, College Station, TX, USA). Values of *P* < 0.05 were considered statistically significant.

## Results

During a median follow-up of 11.6 years, we newly identified 396 LTCI certifications for support or care levels (13.7%), 172 for care levels 2–5 (6.0%), and 162 for support or care levels with dementia (5.6%). We also recorded 307 deaths (10.6%) and 424 composite outcomes of LTCI certifications for care levels 2–5 and death (14.7%). The proportion of participants with any leisure-time physical activity was 54.5% for men and 48.0% for women, and the median amount of leisure-time physical activity in those with any leisure-time physical activity was 18.0 MET-hours/week for men and 13.4 MET-hours/week for women. Men and women with higher amounts of leisure-time physical activity were more likely to participate in this study later, drink more alcohol, participate in social activities more frequently, and walk for longer times, whereas they were less likely to be currently working and in a depressive state (Table 1). Moreover, men who had a higher amount of leisure-time physical activity were more likely to be highly educated and have a history of diabetes mellitus, and less likely to be current smokers. The most common leisure-time physical activities were golf (23.8%), running or jogging (14.4%), and calisthenics (7.9%) in men, and calisthenics (13.9%), running or jogging (10.8%), and swimming (10.3%) in women. In addition, 21.0% of men (n = 303) and 24.6% of women (n = 355) reported activities other than the pre-coded types. Such activities noted in the free comment field included walking (46.9% of all non-pre-coded activities), table tennis (10.9%), and dancing (8.9%).

**Table 1:** Characteristics of the study participants according to amount of leisure-time physical activity.

Variable	Men				Women			
	Leisure-time physical activity (MET-hours/week)			*P* value^a^	Leisure-time physical activity (MET-hours/week)			*P* value^a^
	0.0	0.1–18.0	18.1–261.9		0.0	0.1–13.4	13.5–83.3	
Number of participants	658	403	384		750	347	346	
Year of participation								
1996–2000	325 (50.4)	166 (25.7)	154 (23.9)	0.004	370 (55.0)	162 (24.1)	140 (20.8)	0.024
2001–2005	333 (41.6)	237 (29.6)	230 (28.8)		380 (49.3)	185 (24.0)	206 (26.7)	
Working currently								
No	251 (40.8)	159 (25.9)	205 (33.3)	<0.001	504 (48.1)	257 (24.6)	285 (27.3)	<0.001
Yes	397 (49.0)	241 (29.7)	173 (21.3)		232 (60.9)	89 (23.4)	60 (15.8)	
Missing	10 (52.6)	3 (15.8)	6 (31.6)		14 (87.5)	1 (6.3)	1 (6.3)	
Marital status								
Married	617 (45.1)	385 (28.1)	367 (26.8)	0.58	608 (51.2)	289 (24.3)	291 (24.5)	0.62
Others	37 (51.4)	18 (25.0)	17 (23.6)		132 (54.6)	56 (23.1)	54 (22.3)	
Missing	4 (100.0)	0 (0.0)	0 (0.0)		10 (76.9)	2 (15.4)	1 (7.7)	
Educational attainment								
High school and lower	496 (50.5)	266 (27.1)	221 (22.5)	<0.001	639 (52.9)	284 (23.5)	285 (23.6)	0.099
Junior college and higher	158 (34.6)	136 (29.8)	163 (35.7)		102 (45.1)	63 (27.9)	61 (27.0)	
Missing	4 (80.0)	1 (20.0)	0 (0.0)		9 (100.0)	0 (0.0)	0 (0.0)	
Smoking status								
Never	127 (45.0)	88 (31.2)	67 (23.8)	<0.001	687 (52.1)	320 (24.2)	313 (23.7)	0.076
Former	285 (40.7)	196 (28.0)	220 (31.4)		30 (41.7)	17 (23.6)	25 (34.7)	
Current	246 (53.4)	119 (25.8)	96 (20.8)		33 (64.7)	10 (19.6)	8 (15.7)	
Missing	0 (0.0)	0 (0.0)	1 (100.0)		0 (0.0)	0 (0.0)	0 (0.0)	
Alcohol consumption								
None	275 (50.6)	135 (24.8)	134 (24.6)	0.015	658 (53.5)	295 (24.0)	276 (22.5)	0.003
≤23 g/day (men), current (women)	222 (40.6)	174 (31.8)	151 (27.6)		92 (43.0)	52 (24.3)	70 (32.7)	
23 g/day (men)	161 (45.5)	94 (26.6)	99 (28.0)					
Body mass index								
<18.5	39 (61.9)	16 (25.4)	8 (12.7)	0.052	41 (54.7)	19 (25.3)	15 (20.0)	0.79
18.5 to 24.9	459 (44.3)	290 (28.0)	287 (27.7)		555 (51.2)	261 (24.1)	268 (24.7)	
≥25.0	160 (46.2)	97 (28.0)	89 (25.7)		154 (54.2)	67 (23.6)	63 (22.1)	
Hypertension								
No	317 (43.9)	217 (30.0)	189 (26.1)	0.19	450 (51.1)	212 (24.1)	219 (24.9)	0.58
Yes	341 (47.3)	186 (25.8)	194 (26.9)		300 (53.4)	135 (24.0)	127 (22.6)	
Missing	0 (0.0)	0 (0.0)	1 (100.0)		0 (0.0)	0 (0.0)	0 (0.0)	
Diabetes mellitus								
No	573 (45.9)	361 (28.9)	315 (25.2)	0.010	699 (51.9)	329 (24.4)	318 (23.6)	0.31
Yes	82 (42.9)	42 (22.0)	67 (35.1)		51 (52.6)	18 (18.6)	28 (28.9)	
Missing	3 (60.0)	0 (0.0)	2 (40.0)		0 (0.0)	0 (0.0)	0 (0.0)	
Dyslipidemia								
No	313 (47.7)	171 (26.1)	172 (26.2)	0.24	264 (52.4)	114 (22.6)	126 (25.0)	0.31
Yes	343 (43.6)	232 (29.5)	211 (26.8)		486 (51.8)	233 (24.8)	219 (23.4)	
Missing	2 (66.7)	0 (0.0)	1 (33.3)		0 (0.0)	0 (0.0)	1 (100.0)	
Neuralgia and/or Low back pain								
No	582 (45.1)	367 (28.5)	341 (26.4)	0.72	619 (52.2)	288 (24.3)	278 (23.5)	0.59
Past	33 (50.8)	15 (23.1)	17 (26.2)		51 (47.2)	22 (20.4)	35 (32.4)	
Current	43 (47.8)	21 (23.3)	26 (28.9)		80 (53.3)	37 (24.7)	33 (22.0)	
Geriatric Depression Scale								
≤5	464 (41.7)	325 (29.2)	325 (29.2)	<0.001	502 (47.9)	266 (25.4)	281 (26.8)	<0.001
≥6	170 (60.5)	66 (43.5)	45 (16.0)		192 (59.8)	71 (22.1)	58 (18.1)	
Missing	24 (48.0)	12 (24.0)	14 (28.0)		56 (76.7)	10 (13.7)	7 (9.6)	
Social activity score^b^								
≤25 (men), ≤27 (women)	234 (52.6)	113 (25.4)	98 (22.0)	<0.001	272 (64.2)	75 (17.7)	77 (18.2)	<0.001
26–28 (men), 28–31 (women)	165 (43.0)	123 (32.0)	96 (25.0)		221 (50.6)	111 (25.4)	105 (24.0)	
≥29 (men), ≥32 (women)	236 (40.9)	163 (28.3)	178 (30.9)		210 (41.7)	144 (28.6)	150 (29.8)	
Missing	23 (59.0)	4 (10.3)	12 (30.8)		47 (60.3)	17 (21.8)	14 (18.0)	
Total walking time per day								
<30 min	147 (57.9)	69 (27.2)	38 (15.0)	<0.001	78 (63.9)	29 (23.8)	15 (12.3)	0.002
30 min–1 hour	184 (37.5)	163 (33.2)	144 (29.3)		166 (46.2)	102 (28.4)	91 (25.4)	
1–2 h	143 (38.9)	100 (27.2)	125 (34.0)		204 (49.5)	94 (22.8)	114 (27.7)	
≥2 h	180 (55.1)	71 (21.7)	76 (23.2)		297 (55.1)	119 (22.1)	123 (22.8)	
Missing	4 (80.0)	0 (0.0)	1 (20.0)		5 (45.5)	3 (27.3)	3 (27.3)	

MET, metabolic equivalent.

a Characteristics of participants were compared across groups using the chi-squared test of independence.

b Range: 18–54 points; a higher score indicates more frequent social activity.

### Amount of leisure-time physical activity and incidence of disability

In men, the amount of leisure-time physical activity was inversely associated with the incidence of disability (Table 2). Compared with the no activity group, participants reporting 18.1 MET-hours/week or more of activity had a 52% lower risk of support or care levels with dementia (HR 0.48; 95% confidence interval [CI], 0.25–0.94; *P* for trend = 0.026) in the fully-adjusted model (model 3). Weak inverse associations were also found for support or care levels and for care levels 2–5, but these associations were not statistically significant. In women, no significant associations were found between amount of leisure-time physical activity and incidence of disability (Table 2).

**Table 2:** Associations between amount of leisure-time physical activity and incidence of disability.

	Men							Women						
	Leisure-time physical activity (MET-hours/week)	Person-years	Number of participants	Number of events	Model 1^a^	Model 2^b^	Model 3^c^	Leisure-time physical activity (MET-hours/week)	Person-years	Number of participants	Number of events	Model 1^a^	Model 2^b^	Model 3^c^
					HR (95% CI)	HR (95% CI)	HR (95% CI)					HR (95% CI)	HR (95% CI)	HR (95% CI)
Support or care levels	0.0	6804	658	86	1.00 (reference)	1.00 (reference)	1.00 (reference)	0.0	8004	750	129	1.00 (reference)	1.00 (reference)	1.00 (reference)
	0.1–18.0	4157	403	41	0.81 (0.56–1.17)	0.79 (0.54–1.16)	0.84 (0.57–1.25)	0.1–13.4	3747	347	48	0.79 (0.57–1.10)	0.79 (0.56–1.10)	0.87 (0.62–1.22)
	18.1–261.9	4046	384	34	0.67 (0.45–1.00)	0.70 (0.46–1.06)	0.68 (0.44–1.06)	13.5–83.3	3611	346	58	1.03 (0.75–1.40)	1.04 (0.76–1.43)	1.06 (0.77–1.48)
	*P* for trend				0.042	0.071	0.084	*P* for trend				0.90	0.95	0.85
Care levels 2–5	0.0	6957	658	48	1.00 (reference)	1.00 (reference)	1.00 (reference)	0.0	8327	750	51	1.00 (reference)	1.00 (reference)	1.00 (reference)
	0.1–18.0	4203	403	24	0.85 (0.52–1.38)	0.86 (0.52–1.42)	0.93 (0.56–1.53)	0.1–13.4	3865	347	16	0.68 (0.39–1.19)	0.69 (0.39–1.22)	0.80 (0.45–1.44)
	18.1–261.9	4092	384	15	0.53 (0.30–0.96)	0.54 (0.30–0.99)	0.56 (0.30–1.05)	13.5–83.3	3758	346	18	0.80 (0.47–1.36)	0.85 (0.49–1.47)	0.90 (0.50–1.59)
	*P* for trend				0.036	0.051	0.088	*P* for trend				0.28	0.40	0.62
Support or care levels with dementia	0.0	6975	658	47	1.00 (reference)	1.00 (reference)	1.00 (reference)	0.0	8357	750	47	1.00 (reference)	1.00 (reference)	1.00 (reference)
	0.1–18.0	4210	403	19	0.69 (0.41–1.18)	0.70 (0.40–1.21)	0.72 (0.41–1.27)	0.1–13.4	3888	347	12	0.55 (0.29–1.04)	0.58 (0.30–1.09)	0.62 (0.32–1.19)
	18.1–261.9	4107	384	12	0.44 (0.23–0.83)	0.48 (0.25–0.92)	0.48 (0.25–0.94)	13.5–83.3	3751	346	25	1.24 (0.76–2.01)	1.30 (0.79–2.15)	1.37 (0.82–2.31)
	*P* for trend				0.008	0.019	0.026	*P* for trend				0.66	0.53	0.37
Death	0.0	7104	658	113	1.00 (reference)	1.00 (reference)	1.00 (reference)	0.0	8536	750	45	1.00 (reference)	1.00 (reference)	1.00 (reference)
	0.1–18.0	4265	403	59	0.88 (0.64–1.20)	1.01 (0.73–1.39)	1.11 (0.80–1.55)	0.1–13.4	3929	347	21	1.03 (0.61–1.73)	1.04 (0.62–1.74)	1.09 (0.64–1.86)
	18.1–261.9	4131	384	44	0.67 (0.48–0.95)	0.79 (0.55–1.14)	0.85 (0.58–1.24)	13.5–83.3	3814	346	25	1.28 (0.79–2.09)	1.29 (0.78–2.13)	1.31 (0.78–2.18)
	*P* for trend				0.027	0.26	0.52	*P* for trend				0.35	0.34	0.32
Care levels 2–5 or death	0.0	6957	658	141	1.00 (reference)	1.00 (reference)	1.00 (reference)	0.0	8325	750	86	1.00 (reference)	1.00 (reference)	1.00 (reference)
	0.1–18.0	4203	403	77	0.91 (0.69–1.20)	1.02 (0.77–1.36)	1.12 (0.84–1.50)	0.1–13.4	3865	347	28	0.70 (0.46–1.08)	0.72 (0.47–1.11)	0.81 (0.52–1.25)
	18.1–261.9	4092	384	52	0.63 (0.46–0.86)	0.71 (0.51–0.99)	0.74 (0.52–1.04)	13.5–83.3	3758	346	40	1.05 (0.72–1.53)	1.10 (0.75–1.62)	1.14 (0.77–1.70)
	*P* for trend				0.006	0.071	0.16	*P* for trend				0.93	0.87	0.66

CI, confidence interval; HR, hazard ratio; MET, metabolic equivalent.

a Without adjustment.

b Model 2 was adjusted for year of participation (continuous variable), currently working (yes or no), marital status (married or other [single, divorced, widowed]), educational attainment (high school and lower or junior college and higher), smoking status (never, former or current), alcohol consumption (men: none, ≤23 g/day or >23 g/day; women: none or current drinkers), and body mass index (<18.5, 18.5–24.9, or ≥25.0).

c Model 3 was adjusted for the confounding factors in Model 2 and hypertension (yes or no), diabetes mellitus (yes or no), dyslipidemia (yes or no), neuralgia and/or low back pain (no, past, or current), Geriatric Depression Scale (≤5, ≥6, or missing), social activity score (men: ≤25, 26–28, 29–54, or missing; women: ≤27, 28–31, 32–54, or missing), and total walking time per day (<30 min, 30min–1 hour, 1–2 h, or ≥2 h).

### Sensitivity analysis

When excluding the incidence of disability or death within 3 years from baseline, the association between amount of leisure-time physical activity and disability onset in men remained nearly the same (support or care levels with dementia: HR for 18.1 MET-hours/week or more 0.49; 95% CI, 0.25–0.97; *P* for trend = 0.030 [eTable 1]). When excluding respondents who participated in this study through 1999, many events were dropped, which decreased the statistical power. However, most point estimates of HRs for higher activity remained smaller than unity in the analyses for level of leisure-time physical activity in men (eTable 2). Finally, excluding LTCI certification until March 31, 2002 scarcely changed the number of participants and events or the findings (eTable 3).

## Discussion

The primary finding from the present study was a significant inverse dose–response relationship between the amount of leisure-time physical activity and the incidence of disability with dementia among young elderly males. This finding may be useful to health care providers in recommending leisure-time physical activity for men.

A previous systematic review recommended any type of physical activity for a duration of 150–180 min/week at a moderate to vigorous intensity, with each session lasting 10 min or longer, in order to help prevent disability among the elderly.^40^ Japanese guideline recommended engaging in physical activity of 10 MET-hours/week at any intensity for adults 65 years of age or older.^43^ Nevertheless, these values were estimated using physical activity categories with large variations^40^ or outcomes other than disability (e.g., incidence of osteoporotic fracture and depression).^43^ Therefore, the amount of activity appropriate to prevent disability remains unclear. Because of the small number of events in this study, we may not have determined the appropriate amount of physical activity. However, we identified a dose–response relationship between the amount of leisure-time physical activity and the incidence of disability with dementia in men based on quantitative physical activity assessment, so we can say that more leisure-time physical activity is advisable within the range including 18 MET-hours/week (the median in those with leisure-time physical activity).

Contrary to the findings in men, no associations were found between amount of leisure-time physical activity and risk of disability in women. The relatively lower amounts of leisure-time physical activity among females in our study might have attenuated the effect of activity. In fact, the median amount of leisure-time physical activity among the whole population was 4.4 MET-hours/week in men and 0 MET-hours/week in women. A previous study reported that the percentages of days in a week on which light and heavy housework were done were 66.4% and 63.4% in men and 99.5% and 92.1% in women, respectively.^56^ Although we did not measure physical activity at home, this activity might have more strongly affected the risk of disability in women.

Although physical activity has been shown to be associated with a reduced risk of mortality,^57^, ^58^ we did not find such an association in this study. Arrieta et al reported that frequent leisure-time physical activity was associated with a significant risk reduction of all-cause mortality in older adults, but not in middle-aged adults.^59^ Therefore, the relatively younger age of the participants in the present study may have attenuated the relationship between leisure-time physical activity and mortality.

In the present study, amount of leisure-time physical activity was particularly associated with support or care levels with dementia among men. Recent meta-analyses of cohort studies found that physical activity reduced the risk of cognitive decline^60^ and Alzheimer's disease.^61^ The results in the present study corroborate these findings in the young elderly regarding the risk of dementia with long-term care needs. Some underlying mechanisms have been suggested for the protective effects of physical activity against cognitive decline and Alzheimer's disease, including an increased release of neurotrophins; a reduction in cortisol levels; an increased supply of oxygen and nutrients resulting from increased blood flow; improvement of various cardiovascular risk factors, such as hypertension and diabetes mellitus; and a reduction in the risk of cardiovascular and cerebrovascular disease.^60^

On the other hand, the associations of the amount of physical activity with support or care levels and care levels 2–5 were not significant. Previous studies reported a dose–response relationship between disability regardless of dementia and physical activity as measured using an accelerometer.^32^, ^35^ This inconsistency may have resulted from differences in the measurement of physical activity and/or the small number of events in participants engaging in leisure-time physical activity in our study. Because the amount of leisure-time physical activity was marginally associated with disability regardless of dementia, further follow-up and/or larger studies are warranted.

The unique strength of our study is that all the participants were nearly the same age, which allowed us to eliminate confounding by age. In addition, in this study, we comprehensively collected information on demographic, lifestyle, medical, psychological, and social factors. Therefore, we could adjust for potential confounding factors extensively. However, it should be noted that physical activity may have affected the risk of disability through the improvement of medical conditions. In addition, because the duration of follow-up in the present study (median: 11.6 years) was longer than that of previous studies (e.g., 2–9 years^6^, ^7^, ^8^, ^9^, ^10^, ^11^, ^12^, ^13^, ^15^, ^16^, ^21^, ^23^, ^24^, ^25^, ^27^, ^29^, ^31^, ^32^, ^34^, ^35^, ^37^, ^38^, ^39^), our results may more accurately reflect the long-term effects of physical activity on the risk of disability.

On the other hand, some limitations should be noted. First, we used a self-report measure to assess leisure-time physical activity. Second, we only assessed leisure-time physical activity at baseline; some participants might have subsequently changed their activity patterns. Third, the LTCI system was launched after the start of the present study. If the LTCI system had started before 2000, those who participated in the study from 1996 to 1999 might have received LTCI certification at that time. However, when we excluded LTCI certification within 2 years from the start of the LTCI system, only a few events were lost (sensitivity analysis, eTable 3), so the results were nearly the same. This suggests that the participants who developed disability and were in poor health before 2000, if any, might not have substantially altered the findings. On the other hand, for persons who participated in the baseline survey through 1999, the follow-up duration was underestimated. Therefore, the associations in this study might have been underestimated. Fourth, only part (25%) of the information regarding non-pre-coded activity types was available. Therefore, we estimated a weighted-average intensity (3.9 METs) of items based on the available data, which could have led to some misclassification in the amount of activity.

In conclusion, the findings in this study showed an inverse dose–response relationship between the amount of leisure-time physical activity and disability with dementia among young elderly men. Therefore, a higher level of physical activity may be advisable to young elderly men in order to prevent disability with dementia.

## Financial support

This study was supported by Grants-in-Aid for Scientific Research from the Japanese Ministry of Education, Culture, Sports, Science and Technology (Nos. 15390197, 26460760 and 26520105).

## Conflicts of interest

None declared.
