# Supplementary material for: Leisure-time physical activity and risk of disability incidence: A 12-year prospective cohort study among young elderly of the same age at baseline
Source: J Epidemiol. 2017 Jun 9;27(11):538–45. doi: 10.1016/j.je.2016.11.004 (PMC5608599; doi:10.1016/j.je.2016.11.004)
Supplement: Supplementary file 1 [file mmc1.pdf]

eTable 1. Associations between amount of leisure–time physical activity and incidence of disability, excluding events within 3 years from baseline

	Men					Women				
	Leisure–time physical activity (MET–hours/week)	Person–years	Number of participants	Number of events	Model 4[a] HR (95% CI)	Leisure–time physical activity (MET–hours/week)	Person–years	Number of participants	Number of events	Model 4[a] HR (95% CI)
Support or care levels	0.0	6,790	651	79	1.00 (reference)	0.0	7,987	739	118	1.00 (reference)
	0.1–18.0	4,154	402	40	0.88 (0.59–1.31)	0.1–13.4	3,738	342	43	0.85 (0.59–1.22)
	18.1–261.9	4,043	383	33	0.72 (0.45–1.13)	13.5–83.3	3,606	343	55	1.11 (0.79–1.56)
	P for trend				0.15	*P* for trend				0.71
Care levels 2–5	0.0	6,951	656	46	1.00 (reference)	0.0	8,319	745	46	1.00 (reference)
	0.1–18.0	4,203	403	24	0.95 (0.57–1.60)	0.1–13.4	3,861	345	14	0.79 (0.42–1.47)
	18.1–261.9	4,089	383	14	0.55 (0.29–1.03)	13.5–83.3	3,758	346	18	1.00 (0.56–1.80)
	P for trend				0.083	*P* for trend				0.89
Support or care levels with dementia	0.0	6,971	656	45	1.00 (reference)	0.0	8,351	747	44	1.00 (reference)
	0.1–18.0	4,201	402	18	0.70 (0.39–1.24)	0.1–13.4	3,884	345	10	0.54 (0.26–1.08)
	18.1–261.9	4,107	384	12	0.49 (0.25–0.97)	13.5–83.3	3,751	346	25	1.41 (0.84–2.38)
	P for trend				0.030	*P* for trend				0.34
Death	0.0	7,070	638	93	1.00 (reference)	0.0	8,521	740	35	1.00 (reference)
	0.1–18.0	4,252	394	50	1.15 (0.80–1.66)	0.1–13.4	3,923	344	18	1.19 (0.66–2.13)
	18.1–261.9	4,111	375	35	0.84 (0.55–1.28)	13.5–83.3	3,808	343	22	1.46 (0.84–2.55)
	P for trend				0.56	*P* for trend				0.19
Care levels 2–5 or death	0.0	6,917	636	119	1.00 (reference)	0.0	8,302	735	71	1.00 (reference)
	0.1–18.0	4,190	394	68	1.17 (0.86–1.60)	0.1–13.4	3,855	342	23	0.81 (0.50–1.32)
	18.1–261.9	4,070	374	42	0.71 (0.48–1.04)	13.5–83.3	3,752	343	37	1.28 (0.84–1.96)
	P for trend				0.17	*P* for trend				0.35

CI, confidence interval; HR, hazard ratio; MET, metabolic equivalent.

[a]Excluding participants whose events occurred within 3 years from baseline. The model was adjusted for year of participation (continuous variable), currently working (yes or no), marital status (married or other [single, divorced, widowed]), educational attainment (high school and lower or junior college and higher), smoking status (never, former, or current), alcohol consumption (men: none, ≤23 g/day, or >23 g/day; women: none or current drinkers), body mass index (<18.5, 18.5–24.9, or ≥25.0), hypertension (yes or no), diabetes mellitus (yes or no), dyslipidemia (yes or no), neuralgia and/or low back pain (no, past, or current), Geriatric Depression Scale (≤5, ≥6, or missing), social activity score (men: ≤25, 26–28, 29–54, or missing; women: ≤27, 28–31, 32–54, or missing), and total walking time per day (<30 minutes, 30minutes–1 hour, 1–2 hours, or ≥2 hours).

eTable 2. Associations between amount of leisure-time physical activity and incidence of disability, excluding participants until 1999

	Men					Women				
	Leisure-time physical activity (MET-hours/week)	Person-years	Number of participants	Number of events	Model 5[a] HR (95% CI)	Leisure-time physical activity (MET-hours/week)	Person-years	Number of participants	Number of events	Model 5[a] HR (95% CI)
Support or care levels	0.0	4,022	405	44	1.00 (reference)	0.0	4,628	457	50	1.00 (reference)
	0.1–18.0	2,831	282	22	0.64 (0.37–1.11)	0.1–13.4	2,262	220	15	0.63 (0.34–1.16)
	18.1–261.9	2,759	271	17	0.55 (0.30–1.02)	13.5–83.3	2,387	244	30	1.36 (0.83–2.23)
	P for trend				0.038	*P* for trend				0.34
Care levels 2–5	0.0	4,099	405	22	1.00 (reference)	0.0	4,757	457	19	1.00 (reference)
	0.1–18.0	2,851	282	14	1.02 (0.49–2.10)	0.1–13.4	2,305	220	2	0.25 (0.06–1.09)
	18.1–261.9	2,776	271	10	0.74 (0.33–1.66)	13.5–83.3	2,464	244	9	1.03 (0.42–2.50)
	P for trend				0.51	*P* for trend				0.79
Support or care levels with dementia	0.0	4,103	405	23	1.00 (reference)	0.0	4,764	457	16	1.00 (reference)
	0.1–18.0	2,854	282	10	0.68 (0.30–1.52)	0.1–13.4	2,311	220	0	
	18.1–261.9	2,775	271	8	0.58 (0.25–1.37)	13.5–83.3	2,465	244	13	1.66 (0.71–3.87)
	P for trend				0.18	*P* for trend				0.29
Care levels 2–5 or death	0.0	4,099	405	69	1.00 (reference)	0.0	4,757	457	36	1.00 (reference)
	0.1–18.0	2,851	282	47	1.09 (0.74–1.62)	0.1–13.4	2,305	220	9	0.57 (0.27–1.20)
	18.1–261.9	2,776	271	26	0.66 (0.41–1.06)	13.5–83.3	2,464	244	23	1.33 (0.75–2.36)
	P for trend				0.14	*P* for trend				0.45

CI, confidence interval; HR, hazard ratio; MET, metabolic equivalent.

[a]Excluding participants until 1999. The model was adjusted for year of participation (continuous variable), currently working (yes or no), marital status (married or other [single, divorced, widowed]), educational attainment (high school and lower or junior college and higher), smoking status (never, former, or current), alcohol consumption (men: none, ≤23 g/day, or >23 g/day; women: none or current drinkers), body mass index (<18.5, 18.5–24.9, or ≥25.0), hypertension (yes or no), diabetes mellitus (yes or no), dyslipidemia (yes or no), neuralgia and/or low back pain (no, past, or current), Geriatric Depression Scale (≤5, ≥6, or missing), social activity score (men: ≤25, 26–28, 29–54, or missing; women: ≤27, 28–31, 32–54, or missing), and total walking time per day (<30 minutes, 30minutes–1 hour, 1–2 hours, or ≥2 hours).

eTable 3. Associations between amount of leisure-time physical activity and incidence of disability, excluding events within 2 years from the launch of the long-term care insurance system (March 31, 2002)

	Men					Women				
	Leisure-time physical activity (MET-hours/week)	Person-years	Number of participants	Number of events	Model 6[a] HR (95% CI)	Leisure-time physical activity (MET-hours/week)	Person-years	Number of participants	Number of events	Model 6[a] HR (95% CI)
Support or care levels	0.0	6,802	656	84	1.00 (reference)	0.0	8,002	746	125	1.00 (reference)
	0.1–18.0	4,157	403	41	0.86 (0.58–1.28)	0.1–13.4	3,742	344	45	0.84 (0.59–1.19)
	18.1–261.9	4,046	384	34	0.69 (0.44–1.08)	13.5–83.3	3,611	346	58	1.09 (0.78–1.52)
	P for trend				0.10	*P* for trend				0.76
Care levels 2–5	0.0	6,957	658	48	1.00 (reference)	0.0	8,325	747	48	1.00 (reference)
	0.1–18.0	4,203	403	24	0.93 (0.56–1.56)	0.1–13.4	3,863	346	15	0.81 (0.44–1.47)
	18.1–261.9	4,092	384	15	0.56 (0.30–1.05)	13.5–83.3	3,758	346	18	0.96 (0.54–1.72)
	P for trend				0.088	*P* for trend				0.80
Support or care levels with dementia	0.0	6,973	657	46	1.00 (reference)	0.0	8,356	749	46	1.00 (reference)
	0.1–18.0	4,210	403	19	0.74 (0.42–1.30)	0.1–13.4	3,887	346	11	0.58 (0.30–1.14)
	18.1–261.9	4,107	384	12	0.48 (0.25–0.95)	13.5–83.3	3,751	346	25	1.40 (0.83–2.35)
	P for trend				0.029	*P* for trend				0.35

CI, confidence interval; HR, hazard ratio; MET, metabolic equivalent.

[a]Excluding participants whose events occurred within 2 years from the launch of the long-term care insurance system. The model was adjusted for year of participation (continuous variable), currently working (yes or no), marital status (married or other [single, divorced, widowed]), educational attainment (high school and lower or junior college and higher), smoking status (never, former, or current, alcohol consumption (men: none, ≤23 g/day, or >23 g/day; women: none or current drinkers), body mass index (<18.5, 18.5–24.9, or ≥25.0), hypertension (yes or no), diabetes mellitus (yes or no), dyslipidemia (yes or no), neuralgia and/or low back pain (no, past, or current), Geriatric Depression Scale (≤5, ≥6, or missing), social activity score (men: ≤25, 26–28, 29–54, or missing; women: ≤27, 28–31, 32–54, or missing), and total walking time per day (<30 minutes, 30minutes–1 hour, 1–2 hours, or ≥2 hours).
